# Navigating the Complexities of Intraventricular Hemorrhage in Preterm Infants: An Updated Review

**DOI:** 10.7759/cureus.38985

**Published:** 2023-05-13

**Authors:** Pulliahgaru Apeksha Reddy, Himabindu Sreenivasulu, Mohammad Shokrolahi, Sudheer Kumar Muppalla, Nurlan Abdilov, Rajasekar Ramar, Raghu Halappa Nagaraj, Sravya Vuppalapati

**Affiliations:** 1 Pediatric Medicine, P.E.S. Institute of Medical Sciences and Research, Kuppam, IND; 2 General Medicine, P.E.S. Institute of Medical Sciences and Research, Kuppam, IND; 3 Internal Medicine, University of Debrecen, Debrecen, HUN; 4 Pediatrics, P.E.S. Institute of Medical Sciences and Research, Kuppam, IND; 5 Internal Medicine, Rajah Muthiah Medical College and Hospital, Chidambaram, IND; 6 Surgery, Avalon University School of Medicine, Willemstad, CUW

**Keywords:** intraventricular hemorrhage, microcirculation, cognitive impairment, phenobarbital, drift, neurosurgery, neurological, germinal matrix, neonatal, preterm infants

## Abstract

Intraventricular hemorrhage (IVH) is a type of bleeding that occurs in the ventricular cavity of the brain. In this comprehensive study, we provide a summary of the pathogenesis, diagnosis, and treatment of intraventricular hemorrhage in premature infants. Preterm babies are at high risk of developing IVH because their germinal matrix is not fully developed, making their blood vessels more prone to rupture. However, that is not necessarily the case in all preterm babies as the inherent structure of the germinal matrix makes it more susceptible to hemorrhage. Incidences of IVH are discussed based on recent data which states that around 12,000 premature infants in the United States experience IVH each year. Although grades I and II make up the majority of IVH cases and are frequently asymptomatic, IVH remains a significant issue for premature infants in neonatal intensive care facilities worldwide. Grades I and II have been linked to mutations in the type IV procollagen gene, COL4A1, as well as prothrombin G20210A and factor V Leiden mutations. Intraventricular hemorrhage can be detected using brain imaging in the first seven to 14 days following delivery. This review also shines a light on reliable methods for identifying IVH in premature newborns like cranial ultrasound and magnetic resonance imaging along with the treatment of IVH which is primarily supportive and involves the management of intracranial pressure, the correction of coagulation abnormalities, and the prevention of seizures.

## Introduction and background

Intraventricular hemorrhage, which is more prevalent in extremely preterm infants born before 27 weeks of gestation, is also a significant problem in preterm infants born before 32 weeks of gestation [1]. Depending on the severity of the IVH, these infants may experience both short-term and long-term neurological problems [2]. Clinicians should keep in mind that hereditary factors, modification of the germinal matrix, and impairment of cerebral autoregulation are the primary causes of IVH [1]. Since the early 1980s, there has been a steady decline in the prevalence of IVH, and the widespread use of cranial ultrasonography has made it easier to identify antenatal and perinatal risk factors. More precise visualization of the location and extent of IVH has been made possible by the use of magnetic resonance imaging (MRI) [3].

Modern neonatal care for extremely premature infants aims to reduce mortality and long-term neurological damage [4]. Numerous innovative management strategies, drugs, nursing care approaches, and ventilation techniques continue to develop and adapt to the needs of smaller and sicker children in the neonatal intensive care unit, thereby reducing the incidence of IVH [5]. Different medications such as indomethacin have been used as a prophylactic measure for IVH [3].

Despite advances in obstetric and neonatal care over the past century, IVH remains a major cause of morbidity and mortality in premature infants, with a high incidence in infants born at less than 27 weeks of gestation [6]. In light of this, the goal of this article is to provide a summary of the current knowledge regarding the pathogenesis, diagnosis, and available preventive and treatment strategies for intraventricular hemorrhage in preterm infants.

## Review

Epidemiology

Around 12,000 premature infants in the United States are diagnosed with intraventricular hemorrhage (IVH) annually [7]. The incidence of IVH is negatively correlated with birth weight [8]. The occurrence of IVH in infants weighing less than 1500 g decreased from 40-50% in the early 1980s to 20% in the late 1980s and has remained stable [7]. It is estimated that about 45% of extremely preterm infants are affected by IVH [7]. Intraventricular hemorrhage (IVH) occurs at varying rates in preterm infants, with incidences of approximately 17.0% for grade I, 12.1% for grade II, 3.3% for grade III, and 3.8% for grade IV [9].

After examining trends in hospital admission, death, and comorbidities of newborns with IVH and hydrocephalus over the course of 11 years, despite fewer preterm deliveries, there have been more admissions of preterm infants with IVH, and this rise has been associated with a decrease in the premature mortality rate [10]. Grades I and II account for the majority of IVH cases, and they are typically asymptomatic. The first seven days of life are crucial since the majority of bleeding occurs during this time. Based on a recent study on the incidence of IVH in a limited sample of premature newborns and predisposing factors responsible for it, it was found that 44.44% of harmed neonates survived to adulthood, and 41% of the survivors experienced neurological problems [8]. Consequently, IVH remains a significant issue for premature infants in neonatal intensive care facilities all over the globe as it has significant impacts on the quality of life of preterm infants, leading to developmental delays, cerebral palsy, intellectual disabilities, and other neurological impairments that can have long-term effects. Overall, the burden of IVH is substantial and underscores the importance of preventative measures and effective treatments.

Pathogenesis

The medical condition known as intraventricular hemorrhage can be deconstructed to better understand its meaning. The prefix "intra" means inside, "ventricle" refers to a chamber, and "hemorrhage" means bleeding. Therefore, intraventricular hemorrhage (IVH) refers to bleeding that occurs within a chamber-in this case, the ventricular cavity of the brain, which should contain cerebrospinal fluid rather than blood. Let us examine in greater detail why neonates develop IVH.

Premature infants who have low blood pressure did not receive antenatal corticosteroids, have a low five-minute Apgar score, have higher levels of PaCO_2_ within the first three days of life, and are from multiple pregnancies have a higher likelihood of developing IVH [11]. IVH is also strongly associated with lower gestational age and the use of respiratory support such as continuous positive airway pressure (CPAP). These factors are independent predictors of IVH. To prevent the risk of IVH, it is important to focus on preventing preterm delivery and improving interventions in neonatal care, particularly in early preterm neonates [12].

Preterm babies are at high risk for developing IVH because their germinal matrix is not fully developed, making their blood vessels more prone to rupture. However, that is not necessarily the case in all preterm babies as the inherent structure of the germinal matrix makes it more susceptible to hemorrhage [13]. During the development of the nervous system, the germinal matrix and nearby ventricular germinal zone serve as the location for the proliferation of cortical neuronal and glial precursors [13,5]. The germinal matrix is most noticeable in the region surrounding the head of the caudate nucleus [14]. This region usually has a plentiful blood supply until the fetus reaches maturity [13]. After 24 weeks of gestation, the thickness of the germinal matrix begins to decrease, and it typically disappears around 36-37 weeks. In cases of significant bleeding in the germinal matrix, the underlying ependyma (the lining of the cerebral ventricles) may rupture, causing a hemorrhage to progress to IVH as the blood fills the lateral ventricle [14].

In premature infants with a gestational age of 28 weeks, hemorrhages often occur in the germinal matrix located at the head of the caudate nucleus, specifically at the level of the foramen of Monro. This is a common location for IVH [13,15]. Lacking structural support, the blood vessels in the germinal matrix are more susceptible to rupture [16].

According to Grunnet's research, at the same gestational age, the vasculature in the cortex of human fetuses has smaller luminal areas than the vessels in the germinal matrix. This implies that the wider capillaries in the germinal matrix may be more susceptible to rupture as a result of the increased pressure placed on their walls [17]. The paucity of pericytes decreased fibronectin in the basal lamina and diminished glial fibrillary acidic protein (GFAP) expression in the astrocyte end feet, contributing further to the vulnerability of this region's vasculature [14]. When the germinal matrix bleeds, the underlying ependyma may rupture, leading to IVH.

Genetic factors may have a role in the development of IVH. IVH has been linked to mutations in the type IV procollagen gene, COL4A1, specifically in dizygotic preterm twins [18]. Prothrombin G20210A and factor V Leiden mutations have also been linked to grades I and II of IVH [1].

Diagnosis

Intraventricular hemorrhages can be found using brain imaging in the first seven to 14 days following delivery [19]. Cranial ultrasound (US) can detect IVH and cystic abnormalities [20]. Ultrasound has numerous advantages over computed tomography (CT) and magnetic resonance imaging (MRI), including its safety, portability, availability, and affordability [19]. However, image acquisition is operator- and machine-dependent and restricted by the size of the fontanelle, and less experienced technicians may overlook subtle lesions [19,20]. It has low sensitivity and specificity for detecting diffuse or mild brain damage [20]. These problems collectively may explain why it is challenging to identify white matter injuries using cranial ultrasound images [20].

On the other hand, magnetic resonance imaging (MRI) is a reliable method for detecting subtle hemorrhagic brain lesions such as IVH in premature newborns [20]. It has the highest resolution for identifying low-grade IVH [19]. It allows for a more detailed description of lesions in terms of location and extent compared to cranial ultrasound and computed tomography and offers a more thorough characterization of white matter in newborns [20]. However, MRI is a pricey, time-consuming procedure that is occasionally unavailable and frequently necessitates transportation [19]. Additionally, it is not particularly accessible and is sensitive to movement artifacts [20].

Numerous studies have discovered that significant variations in the measured Doppler perfusion waveform are linked to the subsequent development of IVH [21]. Conventional Doppler ultrasonography, which is the most commonly used modality, has limited sensitivity and can only detect blood flow in large vessels. Additionally, it cannot accurately measure blood flow velocity at a specific point [22]. Ultrafast Doppler imaging, on the other hand, circumvents these limitations by providing highly sensitive angiographic imaging [22].

In one study, the laser Doppler flowmeter showed that infants with low skin blood flow in their feet a couple of hours after birth were at a greater risk of developing IVH [21]. Near-infrared spectroscopy (NIRS) has gained popularity in the study of neonatal cerebral perfusion as it allows for the real-time measurement of parameters related to neonatal cerebral hemodynamics at the bedside [21]. Some authors have found a correlation between the pressure passivity of cerebral perfusion as measured by NIRS and IVH, while others have not [21].

In addition, studies have reported elevated concentrations of various biomarkers in infants with IVH. For instance, infants with IVH have higher levels of S100B in their blood, which is released from astrocytes activated by ischemia [21]. Activin A levels were also found to be elevated in the urine of premature infants who developed IVH [21]. This means that by measuring activin A, we can detect the presence of IVH before the appearance of related ultrasound and clinical signs [23]. Numerous research studies have indicated that the amplitude-integrated electroencephalogram (aEEG) is a reliable method for predicting intraventricular hemorrhage and periventricular leukomalacia [24].

Although newer diagnostic methods and biomarkers seem hopeful in the diagnosis of IVH, more studies are required to demonstrate their value because, despite advancements in monitoring procedures, intraventricular hemorrhage (IVH) and the resulting brain damage continue to be significant complications affecting premature infants [23].

Medical management

The medical community has not yet identified a specific treatment for intraventricular hemorrhage (IVH). The current treatment for IVH is supportive and focuses on preserving cerebral perfusion, minimizing further brain injury, and identifying complications early on [3]. Researchers have proven a few therapies to be successful in preventing complications and improving neurodevelopmental outcomes, but more studies are needed to confirm these findings. The main approach to treating IVH is supportive care, which includes managing intracranial pressure, correcting any coagulation issues, and preventing seizures.

It is hypothesized that the changes in blood pressure, subsequent changes in cerebral blood flow, and oxygen-free radical damage after reperfusion influence IVH. A few clinical trials showed that phenobarbital regulates blood pressure and provides protection against free radical injury but recent studies do not support the use of phenobarbital in managing IVH [25,26].

Indomethacin nonspecifically inhibits cyclooxygenase 1 (COX-1) and cyclooxygenase 2 (COX-2), resulting in decreased prostaglandin synthesis. The use of indomethacin has been shown to improve cerebral autoregulation, enhance microvessel maturation of the germinal matrix, and prevent ischemia-related changes in the blood-brain barrier [26]. Celecoxib, an inhibitor of COX-2, and ZD6474, an inhibitor of the vascular endothelial growth factor R2, both decrease the frequency and severity of germinal matrix bleeding in preterm newborns [6,27]. Although these results are encouraging, more research is necessary before these therapies can be widely implemented in clinical practice.

Surgical management

In some cases, such as in the management of post-hemorrhagic hydrocephalus in premature infants, repeated lumbar punctures or ventricular taps may be used as a short-term measure to alleviate symptoms by preventing the increase of intracranial pressure and reducing the risk of long-term brain damage [28]. Although it is logical to assume that early cerebrospinal fluid (CSF) tapping would lower intracranial pressure and remove blood and protein to clear the CSF routes, Whitelaw and Lee‐Kelland's meta-analysis of four controlled trials found no evidence to support this theory. Whitelaw and Lee‐Kelland also emphasized the risk of secondary infection and the inconvenient nature of the operations [28,29].

Whitelaw and associates tested a technique called drainage, irrigation, and fibrinolytic therapy (DRIFT), in which tissue plasminogen activator (tPA) is administered into the ventricle along with the placement of two ventricular catheters to drain for 72 hours [28,30]. This procedure helps eliminate intraventricular blood and cytokines before hydrocephalus develops [28,30]. Researchers discovered that DRIFT did not affect the number of ventriculoperitoneal shunt operations or deaths among preterm children who had experienced ventricular dilatation after IVH. In addition, the study demonstrated that tapping the ventricular reservoir was a safer and more successful method for treating hydrocephalus following IVH [28,30]. This novel intervention is not advised until additional impartial analyses can offer less ambiguous conclusions [28].

In another procedure called external ventricular drainage, a catheter is inserted into the lateral ventricle, and its proximal end is subcutaneously tunneled into the scalp and connected to a drainage system. External ventricular drainage (EVD) appears to be much more efficient than repeated lumbar punctures (LPs) or ventricular taps for removing enough CSF [28]. Some authors have observed rates of infection with EVD that range from extremely high to really low [31,32].

Prevention

The preventive strategies for intraventricular hemorrhage (IVH) should focus on supporting the germinal matrix vasculature and stabilizing cerebral blood flow (CBF) because IVH is mainly linked to increased vascular fragility and disruption in cerebral blood flow [14]. The use of angiogenic inhibitors, such as glucocorticoids or celecoxib during pregnancy reduces the number of developing blood vessels but does not affect the number of functioning blood vessels that are shielded by pericytes. This results in a stable blood vessel network [14].

Prenatal interventions such as glucocorticoids like betamethasone or dexamethasone are administered to most pregnant women who go into preterm labor with a gestational age of fewer than 34 weeks [33]. Many studies have demonstrated that prenatal glucocorticoids lower IVH incidence and severity [34]. Due to the positive effects of prenatal glucocorticoids, the microvasculature of the germinal matrix is more stable, and there is less disruption of cerebral blood flow [14]. Research has demonstrated that the most favorable outcomes for a premature newborn within one week of delivery can be achieved through prenatal glucocorticoids, specifically with the administration of a full course consisting of either two doses of betamethasone or four doses of dexamethasone [35].

Pregnant women who are in preterm labor should be sent to a tertiary care facility that specializes in high-risk deliveries. Extended labor should be well controlled, given that it may also increase the risk of IVH [14]. Although neonatal resuscitation of preterm newborns is not specifically advised to prevent IVH, it is crucial to restore normal oxygenation and ventilation after birth, as hypoxia and hypercarbia may trigger cerebral blood flow fluctuations that may eventually lead to IVH [14].

Despite the fact that a number of studies suggest that other interventions, such as midline head positioning, may help reduce mortality due to IVH, their efficacy is still unknown. To be more certain about this, high-quality randomized control trials would be required [36]. According to a randomized control trial, the treatment of preterm babies suffering from IVH with recurrent low-dose recombinant human erythropoietin has been shown to yield better outcomes [37].

Phenobarbital and phenytoin are commonly used for seizure prevention in preterm infants which is a common complication of IVH, despite limited efficacy and the risk of potential neurotoxicity. Newer drugs like levetiracetam and topiramate are increasingly used for refractory seizures, although their efficacy, side effect profiles, and pharmacokinetic data are not well-established [38]. Although neonatal treatment has improved, the overall prevalence of IVH has not decreased over the past few decades. Utilizing prenatal glucocorticoids remains the best strategy for preventing IVH [14].

## Conclusions

This review discusses the significant issue of intraventricular hemorrhage (IVH) in very preterm infants, which can cause both short- and long-term neurological problems and describes the pathogenesis, diagnosis, and preventive and treatment strategies for intraventricular hemorrhage in preterm infants. IVH is primarily caused by hereditary factors, changes to the germinal matrix, and impairment of cerebral autoregulation. Diagnostic tools such as cranial ultrasonography and magnetic resonance imaging are useful in identifying antenatal and perinatal risk factors and visualizing the location and extent of IVH. Various management strategies, drugs, nursing care approaches, and ventilation techniques have been developed to reduce the incidence and long-term effects of IVH. Prenatal glucocorticoids, such as betamethasone or dexamethasone, are the most effective way to prevent IVH.
